# Impact of Anxiety on Quality of Life in Parkinson's Disease

**DOI:** 10.1155/2012/640707

**Published:** 2011-11-24

**Authors:** Kristine K. Hanna, Alice Cronin-Golomb

**Affiliations:** Department of Psychology, Boston University, Boston, MA 02215, USA

## Abstract

In Parkinson's disease (PD), both the patient and the health care provider look for ways to preserve the patient's quality of life. Many studies focus on the impact of depression and motor disability on poor life quality but neglect to examine the role of anxiety. We investigated the impact of anxiety and depression on health-related quality of life in PD, using the Parkinson's Disease Quality of Life measure (PDQ-39). Symptoms of anxiety, more than depression, cognitive status, or motor stage, significantly affected quality of life in 38 nondemented patients with mild-to-moderate motor disability. Stepwise regression analyses revealed that anxiety explained 29% of the variance in the PDQ-39 sum score, and depression explained 10% of the variance beyond that accounted for by anxiety. The findings suggest that primary management of anxiety as well as depression may be important to optimizing the quality of life of PD patients.

## 1. Introduction

Parkinson's disease (PD) is a chronic and progressive neurological condition in which nonmotor disturbances as well as motor deficits significantly impact quality of life. The disease is characterized by motor signs including tremor, rigidity, bradykinesia, and disorders of gait and balance. In addition to the difficulties in motor control, which occur as a result of progressive loss of the dopamine-producing neurons in the substantia nigra and dysfunction of the basal ganglia, PD patients also frequently experience disturbances in mood and cognition. These prevalent and disabling nonmotor symptoms may have a greater impact on the patients' quality of life than do the principal motor features of PD [1–6].

Depression is the most commonly explored mood disorder influencing quality of life in PD and has been found to be the best predictor overall for quality of life in several studies [3–7]. In a population-based survey using the Parkinson's Disease Quality of Life Questionniare (PDQ-39) and the Beck Depression Inventory (BDI), Schrag and colleagues [5] found that the factor most strongly related to poorer quality of life was depression, although motor disability was also significantly associated. In a model predicting PDQ-39 scores, the BDI score accounted for 54% of the variance, whereas motor disability scores accounted for only 15%. The Global Parkinson's Disease Survey Steering Committee [6] also found the BDI score to be the most significant predictor of quality of life, accounting for 58% of the variance in PDQ-39 scores, whereas stage of motor severity and PD medication (levodopa, either alone or in combination with other dopaminergic drugs) together explained only 17% of the variability of quality of life in PD. 

Besides depression, anxiety disorders are a clinically significant problem in patients with PD. The prevalence of anxiety has typically been found to be 20–46% of PD patients [8–12] though other studies have reported rates of up to 75% [13]. The number of patients with PD who experience significant anxiety is greater than that of individuals with other chronic medical conditions such as multiple sclerosis or of the general population [13]. Anxiety is thought to have an important impact on motivation, treatment compliance, and cognition and can exacerbate parkinsonian symptoms [14]. 

The contribution of anxiety to quality of life in PD has been less studied, although anxiety symptoms have been found to have a significant association with poorer quality of life in the general population [15]. Most of the few studies that include measures of both anxiety and quality of life have assessed changes in anxiety and quality of life as markers of treatment outcome following a surgical intervention to diminish motor symptoms, rather than directly examining the relation of anxiety to quality of life. One such study by Higginson and colleagues [16] found that improvement in symptoms of anxiety following surgical treatment of PD reflected a true reduction in anxiety as opposed to being simply a reaction to PD motor-symptom amelioration. More recent studies suggest that anxiety contributes to impaired quality of life in PD, using general psychiatric surveys rather than specific anxiety-related instruments [17, 18]. Anxiety symptoms are often comorbid with depression symptoms and the cooccurrence of these disorders is typically characterized by a more chronic course with significant impairment in social and occupational functioning [19]. Improved understanding of the aspects of such symptoms that impact quality of life would lead to increased attention and diagnosis of mood disorders in PD and to formulation of more appropriate treatment plans.

The aim of the present study was to investigate the relation of anxiety and depression to health-related quality of life in PD. Clinical variables that may impact quality of life were explored, including overall cognitive status, disease severity, and side of motor onset, as well as age, education level, and gender. Some studies [16, 20] suggest that certain commonly used measures of anxiety (Beck Anxiety Inventory [BAI]) and depression (Beck Depression Inventory [BDI]) may inflate the prevalence of anxiety and depression symptoms in PD because of the overlap of somatic symptoms of anxiety and depression with disease-related motor symptoms of PD such as trembling or wobbliness in the legs. For this reason, the present study included the Spielberger Anxiety Inventory-Trait (STAI) and the Geriatric Depression Scale (GDS), which are two self-report measures that include fewer somatic items [21–23], in addition to the BAI and BDI. The intention was to establish PD compromise on mood measures without somatic load before concluding that mood itself was directly related to quality of life [16, 20, 24]. We hypothesized that anxiety symptoms, like depression, would predict quality of life more strongly than would motor symptoms. We further expected to find anxiety to uniquely contribute to quality of life in PD.

## 2. Methods

### 2.1. Participants

Thirty-eight nondemented individuals (20 men, 18 women) participated in the study. All participants were recruited from the Boston Medical Center Neurology Clinic and from local PD support groups. Each participant's medical record was reviewed to confirm the diagnosis of idiopathic PD. Informed consent was obtained from each participant. No individual had undergone surgery affecting the thalamus, basal ganglia, or other brain regions. Motor disability was staged using the Hoehn and Yahr scale [25]. At the time of testing, the motor response was at its optimum (“on” period).

 The widely used Mini-Mental State Examination (MMSE [26]) was administered as a brief screen for dementia, which allows us to compare directly the mental status of our sample to those reported in the literature. An MMSE score of 25 or better, indicating nondemented status, was required to participate. Participants were questioned about psychiatric history and current psychotropic medication.

### 2.2. Measures

The Dementia Rating Scale (DRS [27]) assesses cognitive functioning in five domains: attention, initiation/perseveration, construction, conceptualization, and memory. The DRS was chosen as the cognitive status variable because it is a widely used and accepted measure for the assessment of neurocognitive functioning in the geriatric population and it has demonstrated good validity in PD patients [28]. 

The Hoehn and Yahr scale (H/Y) is a standard clinical index of PD motor stage. It globally indexes signs and symptoms of functional impairment, including postural instability, rigidity, tremor, and bradykinesia. Stage I indicates unilateral motor involvement. Stages II and III indicate mild and moderate bilateral disability, respectively. None of our participants was categorized as higher than Stage III (see Section 3).

The 21-item Beck Anxiety Inventory (BAI [29]) assesses anxiety in normal populations. A higher overall score indicates more symptoms of anxiety. The BAI consists of items that represent physiological symptoms such as numbness and tingling, dizziness, and sweating, and items representing “subjective” symptoms of anxiety such as fear of the worst happening, fear of losing control, or feeling scared [29] The Spielberger Anxiety Inventory-Trait (STAI-T [30]) is a 20-item self-report instrument designed to assess trait anxiety. The items are summed to produce a total score, with higher scores indicating more trait anxiety. The STAI-T was included as a measure of anxiety because it does not include somatic items. The STAI-T was developed from the premise that anxiety is the affective response when there is a perceived or actual discrepancy between external demands and coping resources and focused less on the physiological symptoms of anxiety. 

The Beck Depression Inventory II** (**BDI-II [31]) was administered to assess depression symptomatology. The test contains 21 items, most of which assess depressive symptoms on a Likert scale of 0–3. The BDI-II includes 13 items that pertain to somatic symptoms of depression. The Geriatric Depression Scale (GDS [32]) is a 20-item measure of depression that was developed for the geriatric population and omits questions regarding sleep and appetite disturbance, which are symptoms that may result from PD motor symptoms or side effects of PD medication rather than reflecting the presence of depression in PD. Only two items may capture somatic symptoms (energy; restless and fidgety). Conservative cut-off scores were used with the Beck measures when determining the percentage of the sample to have clinically significant levels of anxiety and depressive symptoms [16, 20].

The Parkinson's Disease Quality of Life Questionnaire (PDQ-39 [33]) is a disease-specific, 39-item questionnaire on the quality of life in PD. Quality of life is a multidimensional concept that reflects a patient's subjective evaluation of well-being, satisfaction, functioning, and impairment [34]. Dimensions assessed include mobility, activities of daily living, emotional well-being, stigma, social support, cognitive impairment (arousal, concentration, memory, and dreaming/hallucinations), communication, and bodily discomfort. Lower scores on the PDQ-39 indicate greater quality of life. Summing all eight of the PDQ-39 dimensions and standardizing the score on a scale of 0–100 creates the summary index score of the measure (PDQ-39SI). The PDQ-39SI provides insight into the overall impact of the illness as measured by each of the domains included. The PDQ-39 is the most widely used disease-specific health status questionnaire in the literature and has demonstrated high reliability and validity in PD [33].

### 2.3. Scoring and Statistical Analysis

The summary index of the PDQ-39 was calculated according to the standard scoring algorithm [35]. All variables were converted to standardized *z* scores. Spearman rank correlation coefficients were calculated to assess the direction and magnitude of association between variables. A stepwise hierarchical regression analysis was performed, with variables entered into the regression model in the order of interest (anxiety first, then depression) in accordance with information provided by our preliminary findings [36]. Mann-Whitney *U* test analyses were conducted to determine if side of motor symptom onset, disease duration, age at onset, current age, or gender were associated with the extent of anxiety or depression symptoms.

## 3. Results

### 3.1. Descriptive Analysis

Thirty-eight participants with idiopathic PD were evaluated (Table 1). Participants included two in Stage I (unilateral), 27 in Stage II (mild bilateral), and nine in Stage III (moderate bilateral). For 22 of the participants, motor symptom onset was on the left body side, for 15 onset was on the right side, and for one onset was reported to be bilateral. 

Twelve participants followed a medication regimen that included a combination of levodopa/carbidopa therapy alone (*n* = 2) or in combination with one other dopamine agonist (pramipexole (*n* = 3), pergolide (*n* = 1), or ropinirole, (*n* = 2)). Five participants were treated with levodopa/carbidopa therapy and the catechol-O-methyltransferase inhibitor entacapone, two participants were treated with this regimen (levodopa/carbidopa plus entacapone) and dopamine agonists, and one with this regimen and the anticholinergic trihexyphenidyl. Six participants were treated with trihexyphenidyl plus either levodopa/carbidopa (*n* = 1) or the dopamine agonist pramipexole (*n* = 1). Nine participants received levodopa/carbidopa therapy in combination with additional dopaminergic medications and either the monoamine oxidase type B inhibitor selegiline (*n* = 3) or amantadine (*n* = 1), which stimulates dopamine release. Six individuals were being treated with a dopamine agonist alone and either selegiline (*n* = 1), trihexyphenidyl (*n* = 1), or amantadine (*n* = 1). 

The mean PDQ-39 score was 262.8 (standard deviation 126.3), mean MMSE was 29.0 (1.3), and mean DRS was 141.7 (2.6). The mean depression scores on the BDI-II and GDS were 10.6 (7.0) and 9.3 (6.7), respectively. The mean anxiety score was 14.2 (8.1) on the BAI and 37.0 (10.6) on the STAI-T. Because of the issue of somatic items, we followed the recommendation for higher cut-off scores for each Beck measure in PD [16, 20], which provides greater specificity albeit with decreased sensitivity. Eleven participants (29% of the sample) had clinically significant anxiety when evaluated with the BAI using a highly specific cut-off score of 18 [16] compared to 12 (32%) determined by the STAI-T cut-off score 42. Clinically significant depression was present in eight (21%) when determined by the BDI-II with a highly specific cut-off score of 17 [16] and in 17 (45%) when measured by the GDS with a standard cut-off score of 10, which has yielded 100% sensitivity and 84% specificity for a diagnosis of major depression in primary care settings [37]. 

Patients on antidepressants were all stable on their medication for at least six months and did not differ on their mood scores from those not on antidepressants. A total of ten participants were taking antidepressant medication, which included either paroxetine or sertraline. Despite the medication, two participants still had clinically significant anxiety, three had clinically significant depression and anxiety, and two had clinically significant depression, leaving three without significant levels of anxiety and depression with the psychiatric medication.

Comorbidity of anxiety and depression can be a concern; we used the Beck measures because they have demonstrated good divergent validity in regard to detection of anxiety and depression. Six participants (16% of the sample) had clinically significant anxiety without clinically significant depression, three participants (8%) had clinically significant depression without clinically significant anxiety, five (13%) had both clinically significant anxiety and depression, and 24 (63%) did not have clinically significant anxiety or depression. Hence, there were more participants with principally more anxiety than depression in this sample. 

The percentage of anxiety and depression in the present sample did not differ for men and women, nor for those with motor symptom onset on the right versus the left side of the body (Table 2), nor did there appear to be any interaction of gender and side of onset. No significant differences were found between the gender or side of onset subgroups on quality of life. Mann-Whitney *U* test analyses revealed that side of motor symptom onset, age at onset, and gender were not associated with the extent of anxiety or depression symptoms. Data were accordingly collapsed across these variables for subsequent analyses.

### 3.2. Correlation Analysis Results

Motor symptom stage, age, education, duration of disease, anxiety, depression, and overall cognitive status are variables that have previously been shown to affect health-related quality of life and were included in the correlational analysis (Table 3). The PDQ-39 summary index score correlated significantly and positively with anxiety as measured by the BAI (*ρ* = .54, *P* < .0001) and the STAI-T (*ρ* = .65, *P* = .002) and with depression as measured by the BDI-II (*ρ* = .54, *P* < .0001) and the GDS (*ρ* = .43, *P* < .0001). The PDQ-39 was inversely related to overall cognitive status (DRS) (*ρ* = −.33, *P* = .005), though it should be noted that none of the participants met the criteria for dementia, including scores on the DRS or MMSE. Those suffering from more symptoms of anxiety and depression demonstrated poorer quality of life. Age of disease onset (*ρ* = −.16, *P* = .35), current age (*ρ* = −.04, *P* = .81), duration of disease (*ρ* = .17, *P* = .32), education (*ρ* = −.11, *P* = .51), and motor stage (H/Y score; *ρ* = −.10, *P* = .55) did not correlate significantly with quality of life. Years of education was inversely correlated with symptoms of anxiety as measured by the BAI (*ρ* = −.43, *P* = .007) but not the STAI-T (*ρ* = −.27, *P* = .11) or symptoms of depression (GDS, *ρ* = −.22, *P* = .19; BDI-II, *ρ* = −.17, *P* = .31). 

### 3.3. Multiple Regression Analysis Results

Using multiple regression, the quality of life summary score was then regressed on the linear combination of variables suggested by the literature to impact quality of life including anxiety symptoms (BAI), depression symptoms (BDI-II), motor stage (H/Y), and cognition (DRS). The equation accounting for these four variables accounted for 49% of the variance in quality of life (*F*(4,38) = 9.92, *P* < .001, adjusted *R* ^2^ = 0.49). Because of the good divergent validity of the Beck measures for detecting symptoms of anxiety and depression and our desire to be as conservative as possible in designation of clinical anxiety and depression, we used the Beck measures rather than the STAI-T or GDS for the regression analyses. This decision was supported by our finding that the STAI-T correlated significantly with the GDS (*r* = .79, *P* = .000). 

Only variables that correlated significantly with the PDQ-39 quality of life measure were entered into the stepwise multiple regression analysis. These variables included the anxiety score (BAI), the depression score (BDI-II), and the overall measure of cognitive status (DRS). Anxiety and depression demonstrated nearly equivalent beta-weights with the BDI-II at 0.37 (*t* = 2.39, *P* < .05) and BAI at 0.34 (*t* = 2.23, *P* < .05). The beta-weight for overall cognition (DRS) was −0.24 (*t* = 1.78, *P* > .05), which indicated that cognition did not significantly contribute to the model. All coefficients were in the predicted direction. Overall, anxiety symptoms accounted for 29% of the variance in quality of life beyond the variance accounted for by the other predictors. Following the variance accounted for by anxiety, depression symptoms uniquely accounted for an additional 10% of the variance in quality of life.

## 4. Discussion

We found that symptoms of anxiety, more than depression, overall cognitive status, or motor stage, affect health-related quality of life for nondemented patients with PD. The hypothesis that anxiety symptoms would significantly explain variance in overall quality of life (PDQ-39) in PD was supported, in that anxiety explained 29% of the variance beyond that explained by the other clinical variables in the model. Depression explained an additional 10% of the variance not accounted for by anxiety. Together, these mood symptoms accounted for 39% of the variance in quality of life. Although cognitive status (DRS scores) correlated significantly with quality of life scores, it did not explain any further variance when anxiety and depression were in the model, and in any case none of the participants met criteria for dementia. 

Earlier studies describing quality of life in patients with PD did not include both anxiety and depression in the model. Without including anxiety scores, the majority of these studies found either depression [2–5, 7] or disease severity [3–5] to be the most frequent associate of quality of life. Some previous studies have shown cognitive status, as measured by MMSE, to be an important predictor of quality of life in PD [5], but others have not [4]. 

Studies have found depression to explain up to 50% of the variance in PDQ-39 scores [1, 3, 5, 38]. The current study found depression symptoms to predict only 10% of the variance once anxiety was accounted for. Although differences in sample sizes from study to study may underlie some differences in the size of the contribution of depression, it may also be argued that, if previous studies had included an anxiety score in their model, their results may have been more similar to those of the present study. It is also possible that, because depression and anxiety symptoms often occur together in PD (e.g., [10]), the measures used to capture anxiety and depression are not able to differentiate these two conditions, or an interaction between the two is present. Arguing against this interpretation is the fact that the Beck measures used in the present study have demonstrated good divergent validity between anxiety and depression [39]. 

In spite of the relatively high mean DRS scores for the sample, cognitive status was significantly associated with quality of life, consistent with Schrag and colleagues [5] even though it did not significantly contribute to the model once the mood measures were included. A sample comprising a larger range of cognitive impairment related to PD, including dementia, may show a greater impact of cognitive status on reported quality of life. It should also be noted that brief screening measures such as those used here do not capture more subtle cognitive changes that could contribute to patients' reported well-being. Future studies with tests that assess specific cognitive domains may not only further elucidate the relation of cognition to quality of life but also help determine whether various types of cognitive difficulties—for example, executive functioning versus attention—differentially impact reported quality of life in PD.

We did not find a relation between overall quality of life and motor stage, as measured by the Hoehn and Yahr (H/Y) scale. A study by Hobson and Meara [40] did not find any significant correlation between motor stage, using the H/Y scale, and scores of the SF-36, the Short Form health related quality of life scale, whereas a study by the Global Parkinson's Disease Research Committee [6] found H/Y scores and medication to explain up to 17.3% of the variance in the PDQ-39. In our recent study, we found correlations between only some aspects of motor severity as indexed by the UPDRS and some of the PDQ-39 subscales: specifically, the Rigidity and Dopamine-dependent subscales of the UPDRS with the ADL subscale of the PDQ-39; the Rigidity and Facial Expression subscales of the UPDRS and the Communication subscale of the PDQ-39 [41]. A sample with a wider range of motor severity than represented in our present sample may well yield different results.

Anxiety is prevalent in PD, and this study highlights the dramatic impact it has on quality of life. Despite using conservative cut-off scores and using measures with few somatic items, clinically significant anxiety occurred in one-quarter to one-third of the participants of this study, depending on the measure used. Exactly how anxiety impacts quality of life has yet to be clearly determined. Anxiety symptoms are more prevalent in PD patients than in the general population or in individuals with other chronic illnesses but are not primarily a psychological reaction to the illness or side effects of levodopa treatment [42]. Some investigators suggest that people with anxiety and people with PD share an underlying biological vulnerability. Anxiety and affective symptoms have been associated with striatal dopamine transporter (DAT) availability in the basal ganglia [43] as well as with PD-related loss of catecholinergic cells of the locus ceruleus [12, 13] and abnormalities of serotonin production [13]. It is of substantial interest that mood disorders may precede the onset of PD motor symptoms by several years—even 20 years in the case of anxiety—suggesting that mood disorders may be prodromal indicators of PD [44, 45]. Besides anxiety itself, an anxious (neurotic) personality has also been revealed as a risk factor for PD much later in life [46], again suggesting a common pathophysiology for anxiety disorders and PD.

A more direct relation between anxiety symptom amelioration and improvement in PD symptoms may exist than previously recognized. Anecdotal evidence coupled with a study by Knight et al. [42] suggests that motor symptoms themselves may be influenced by anxiety in PD. For example, the disabling motor symptoms such as tremor, rigidity, bradykinesia, and postural instability, which often occur intermittently, have been shown to increase when the patient is concentrating or feeling anxious [10]. Further research exploring the direct relation of anxiety and motor symptoms and subsequent quality of life needs to be conducted. It is possible that treatment of anxiety in PD not only will increase the perceived quality of life but also may help reduce the frequency of the motor symptoms associated with the disease. 

With 39% of the variance in quality of life accounted for by anxiety and depression symptoms, this study highlights the importance of treating anxiety symptoms as a means to improving the well-being of patients with PD as well as the importance of continued emphasis on the impact of depressive symptoms on their overall well-being. Some investigators have suggested a more pronounced increase in comorbidity of anxiety and depression in patients with PD than in healthy adults (19.3% comorbidity in PD versus 8.6% in control adults) [47]. When depression and anxiety occur together, they are associated with increased impairment, a more chronic course, and poorer outcome, rendering treatment more complex [48–50]. In PD, investigators have suggested that anxiety often presents before the onset of comorbid depression [51]. The strong comorbidity between generalized anxiety disorder and major depression, the fact that most people with this type of comorbidity report that the onset of generalized anxiety disorder occurred before the onset of depression, and the fact that primary generalized anxiety disorder significantly predicts the subsequent onset of depression and other secondary disorders raise the question of whether early intervention and treatment of primary anxiety would effectively prevent the subsequent onset of secondary depression as well as improve PD patients' quality of life.

Limitations of this study include the use of patient self-report and the sample size. Future research can address this issue by including more participants and adding measures of participants' dispositional characteristics. The use of a brief self-report measure of anxiety and depression, although practical, does not allow us to clarify the nature of the anxiety and depressive disorder in this population or to determine whether the specific type of anxiety disorder has a differential impact on quality of life—for example, generalized anxiety disorder versus social phobia versus panic disorder, though all have been associated with PD [11, 52–54]. Replicating this study with a clinician-based interview would help elucidate whether certain types of anxiety disorders have greater impact on quality of life and more confidently clarify the frequency of general anxiety disorder and major depressive disorder in PD. It would also be worthwhile to include a more sensitive measure of PD motor symptoms, such as the UPDRS. In a larger sample, it would be of interest to examine data on disease characteristics associated with anxiety, such as motor fluctuations and dyskinesias. Although the results of this study indicate that mood symptoms are associated with reduced quality of life in patients with PD, this study is cross-sectional and the direction of causation among the variable examined cannot be determined. Longitudinal studies would help clarify the causal relation between the variables in this study and help us to assess how quality of life may change following treatment of anxiety and depression in this population. 

A better understanding of the factors that have the greatest impact on a patient's well-being is important to informing new and improved treatment management plans in PD. The findings of the present study support those of other studies in the literature that mood symptoms are better predictors of quality of life than is motor symptom stage [1–3, 5]. The primary difference between this study and previous studies is the inclusion of standard anxiety measures here and the finding that anxiety, in addition to depression, is associated with quality of life using a PD-specific quality of life measure. 

Despite the prevalence of anxiety and depression in PD, mood symptoms are often not addressed in individuals with this disease. In a chronic and disabling illness such as PD, improving the aspects of well-being that most significantly impact their perceived quality of life is vital [12]. Empirical studies confirm that cognitive behavioral therapy (CBT) is an effective form of therapy for the treatment of anxiety and depression in the general population [55, 56]. Dobkin et al. (1997) demonstrated the effectiveness of CBT for the treatment of depression in PD [57], but further studies are needed to also address the effectiveness of CBT and other therapies for the treatment of anxiety in PD. The findings of the present study suggest that primary assessment and management of the anxiety and depression associated with the disease may be needed to optimize the quality of life of patients with PD, and we accordingly call for more clinical work and research in this area. As there is no cure for PD, empirically supported treatment of distressing neuropsychiatric symptoms is of paramount importance in the quest to improve the quality of life of individuals with this disorder.

## Figures and Tables

**Table 1:** PD participant characteristics; means (SD).

*N*	38
Age, years	62.1 (8.7)
Education, years	16.3 (2.9)
MMSE (total)	29.0 (1.3)
Men:women	20 : 18
Disease duration, years	8.4 (6.4)

MMSE: Mini-Mental State Examination.

**Table 2:** Anxiety and depression symptoms in PD subgroups by gender and side of motor symptom onset.

	Male (*n* = 20)	Female (*n* = 18)	LPD (*n* = 21)	RPD (*n* = 16)
BDI-II	11.8 (7.3)	9.2 (6.5)	10.3 (7.4)	11.0 (6.8)
GDS	10.0 (6.2)	8.6 (7.3)	9.8 (7.6)	9.0 (5.7)
BAI	15.0 (7.4)	13.2 (9.0)	15.1 (9.2)	13.1 (6.9)
STAI-T	38.1 (7.9)	35.8 (13.1)	37.6 (11.4)	36.8 (9.9)

BDI-II: Beck Depression Inventory; GDS: Geriatric Depression Scale; BAI: Beck Anxiety Inventory; STAI-T: Spielberger Trait Anxiety Inventory; LPD: left motor symptom onset PD; RPD: right motor symptom onset PD.

**Table 3:** Spearman correlation matrix for variables previously demonstrated to affect health-related quality of life in PD.

	STAI-T	BDI-II	GDS	DRS	H/Y	AGE	EDU	DUR	PDQ-39
BAI	.68**	.54**	.43**	−.33*	−.31	0.05	−.43**	.19	.54**
STAI-T		.78**	.79**	.23	.13	.05	−.27	−.01	.65**
BDI-II			.79**	−.20	.09	.17	−.17	−.02	.54**
GDS				−.02	−.08	.09	−.22	.08	.43**
DRS					−.37*	−.28	.17	−.24	.33*
H/Y						.16	−.005	.21	−.10
AGE							−.09	.33*	−.04
EDU								−.09	−.11
DUR									.17

*P < .05; **P < .01.

BAI: Beck Anxiety Inventory; STAI-T: Spielberger Trait Inventory; BDI-II: Beck Depression Inventory II; GDS: Geriatric Depression Scale; general cognitive status: DRS: Dementia Rating Scale; H/Y: Hoehn and Yahr index of motor symptom stage; AGE: age at time of testing; EDU: years of education; DUR: years of disease duration.
